# Decreased health-related quality of life in angiodysplasia patients: A cross-sectional cohort

**DOI:** 10.1371/journal.pone.0177522

**Published:** 2017-05-26

**Authors:** Karina V. Grooteman, Mijntje Matheeuwsen, Erwin J. M. van Geenen, Joost P. H. Drenth

**Affiliations:** Department of Gastroenterology and Hepatology, Radboud University Medical Center, Nijmegen, The Netherlands; University Hospital Llandough, UNITED KINGDOM

## Abstract

Gastrointestinal angiodysplasias may cause anemia. Quality of life (QoL) is a valid patient reported outcome and improvement of QoL represents an important treatment goal. There is a paucity of data on the effect of angiodysplasias on QoL. Therefore, we aim to evaluate QoL and fatigue in angiodysplasia patients. We performed a cross-sectional patient-reported outcome study. We included patients with endoscopy proven angiodysplasias and measured QoL with Short Form-36 and level of fatigue using Multi Fatigue Inventory-20. We distinguished three subgroups of patients according to disease severity: 1) with treatment for angiodysplasias, 2) without treatment for angiodysplasias and 3) without recent hospital visits. The primary outcome was the physical component summary (PCS) score on the SF-36. Multivariate regression analysis were performed to correct for differences at baseline. A total of 144 patients completed the questionnaires (response rate = 62%; mean age 68 years; 65% men). Angiodysplasia patients have a significant lower PCS compared to the age-matched general population (respectively 41.0 vs. 43.3, *p* = 0.01). Disease severity is independently associated with a negative outcome on QoL (ß -4.6, 95% CI -7.8–-1.3). Similarly patients score lower on multiple QoL subdomains, i.e. role limitations due to physical health problems (40.8 vs. 44.0, *p*<0.01), general health (39.7 vs. 47.3, *p*<0.01). Angiodysplasia patients are more fatigued compared to the general population (male 56.1 vs. 48.5, *p*<0.01, female 59.2 vs. 51.5, *p* = 0.01). In conclusion, angiodysplasias are independently associated with clinically significant impairments in multiple domains of health-related QoL, especially in measures of functional limitation.

## Introduction

Angiodysplasias are defined as abnormally dilated mucosal veins that portend gastrointestinal (GI) bleeding. The prevalence of GI angiodysplasias in the general population is unknown, but it is seen in around 2% of the population subjected to colonoscopy [1]. Known risk factors are higher age, aorta stenosis, chronic kidney disease and Von Willebrand disease [2]. The spectrum of the angiodysplasia disease phenotype is wide and ranges from absence of symptoms to chronic anemia with frequent red blood cell transfusions and hospitalizations. Anemia is associated with adverse outcomes including functional decline, disability, morbidity and mortality [3].

Conventionally, angiodysplasia related bleeding and associated morbidity are used to define clinical outcomes: i.e. hemoglobin levels, rebleeds, need for endoscopic intervention [4, 5]. Intuitively, improvement of anemia as a response to endoscopic intervention might confer benefits for patients but measuring hemoglobin levels does not necessarily capture how patients actually feel and can be seen as a surrogate outcome. Patient reported outcomes (PRO’s) are multidimensional, unique indicators of the impact of disease on an individual patient [6]. Health related quality of life (QoL) is a PRO which represents the patient’s general perception of the effect of illness on various physical and social aspects of life. It is increasingly recognized that restoration of decreased QoL is an important treatment goal and bonafide outcome in clinical research [7]. There is a paucity of literature on QoL in angiodysplasia patients, but it is known that chronic anemia decreases QoL and that severe fatigue specifically impacts QoL in anemic patients [8].

Based on the prevalence of anemia and the tendency of angiodysplasia bleeding to recur, we hypothesize that angiodysplasia patients will have a reduced health-related QoL and higher levels of fatigue compared to the general population. Furthermore, we surmise that there is a relation between angiodysplasia disease severity and QoL. The primary objective of this study is to investigate health related QoL and fatigue in angiodysplasia patients in a cross-sectional patient-reported outcome study. Our secondary objective is to evaluate possible differences in QoL between angiodysplasia patients who received treatment and those who did not.

## Methods

### Study design and setting

We performed a cross-sectional patient-reported outcome study at the department of Gastroenterology and Hepatology of the Radboud University Medical Center Nijmegen, the Netherlands (S1 Table). Patients with endoscopy confirmed angiodysplasias were recruited into a longitudinal observational cohort between January 2010 and March 2015, with a follow-up until July 2016. We administered validated QoL and fatigue questionnaires in August and September 2016.

### Participants

Inclusion criterion were age ≥ 18 years and one or more proven angiodysplasias, defined by the attending endoscopist reporting the term ‘angiodysplasia‘, ‘angioectasia‘, ‘ectasia‘, ‘telangiectasia‘ or ‘arteriovenous malformation‘ in reports of esophagogastroduodenoscopy, colonoscopy, balloon assisted enteroscopy or video capsule endoscopy. Three subgroups of patients were distinguished to reflect disease severity: 1) treatment in the year prior to the questionnaires, 2) without treatment in the year prior to the questionnaires, 3) without hospital visits in the year prior to the questionnaires. The group with angiodysplasia patients without treatment was further classified according to presence of anemia (defined as Hb <8.5 mmol/L for men and <7.5 mmol/L for women). Subjects were required to read and understand Dutch language to complete the two patient self-assessment questionnaires. Exclusion criteria were cognitive impairment (e.g. severe dementia) or physical inability to complete the questionnaires. The questionnaires were sent via e-mail or regular mail, depending on patient’s preference. There were at least two attempts to contact eligible patients and one reminder in case of no response.

### Outcomes

Based on the hypothesis that QoL in angiodysplasia patients will be determined by physical rather than mental limitations, the primary outcome is the physical summary score (PCS) of the SF-36. Secondary outcomes are subdomain scores of the SF-36 and fatigue assessed by the MFI-20. The eight subdomains are: physical functioning (PF), role limitations due to physical health problems (RP), bodily pain (BP), general health (GH), vitality (VT), social functioning (SF), role limitations due to emotional problems (RE), and mental health (MH).

### Measurements

Baseline measurements including patient and disease characteristics were extracted from the electronic patient report system. Two patient self-assessment questionnaires, the SF-36 and MFI-20, were used to measure the health related QoL and level of fatigue respectively [9, 10]. Two overall scores can be generated from the eight subdomains of the SF-36: the PCS and mental summary score (MCS). A higher score on the 0–100 scale corresponds to a better QoL. The SF-36 questionnaire has been extensively validated in several populations [11–13].

The MFI-20 has shown to have a good reproducibility and internal consistency among different patient populations [10, 14, 15]. Construct validity was demonstrated to be sufficient, by comparing MFI-20 to several other questionnaires such as the SF-36 and the Fatigue Assessment Scale [15, 16]. The MFI-20 consists of 20 items each having a 5-point Likert scale. A higher score reflects a higher level of fatigue.

### Sample size calculation

Based on previous literature the mean age in our study was ought to be >65 years [17]. Therefore, the mean PCS for the general population with age 65–74 years (PCS = 43) was used [18]. As there is no previous literature on QoL in angiodysplasia patients, the effect size was based on studies in patients with chronic kidney disease and anemia [8, 19]. With a power of 0.95, a level of significance of 0.05 and a mean score on the PCS of 38 (compared to 43) with a SD of 10, the sample size was ought to be 88 patients.

### Statistical analysis

Descriptive statistics were used to describe patient and disease characteristics. Categorical variables are expressed as percentages and continuous variables are expressed as mean ± standard deviation (SD) or as median with range for non-normal distributed variables. Differences in baseline characteristics were investigated with Chi-square testing.

To deal with missing data in the SF-36, we used personal mean score imputation of subdomain means, justified for subdomains missing < 50% [20]. Missing questions in the MFI-20 where imputed with the contrary formulated question. When there were >50% missing for the SF-36 or missing the contrary question for the MFI-20, the patient was excluded from analysis.

We used United States of America (USA) population norm values for the SF-36 and German population norm values for the MFI-20 both corrected for age (age group determined based on mean age of our included patients) [21]. Previous studies justify the use of USA norm values for the Dutch population on the SF-36 [22]. One-sample t-tests were used to compare these general population norm values with the study population to assess our primary aim. One-way independent ANOVA with post-hoc Tukey testing was used to compare the PCS, subdomain scores and level of fatigue between the three subgroups of patients: 1) treatment, 2) non-treatment, and 3) patients without hospital visits in the past year. This statistical method was also used to investigate the differences on PCS between treated patients and anemic / non-anemic patients in the non-treated group.

Finally, we performed multivariate linear regression analysis to correct for baseline differences (i.e. comorbidities) between the subgroups for the primary outcome. QoL is strongly correlated with age and a small (not statistical significant) difference between groups could be of influence. Therefore, we included age in the adjusted analysis for the primary outcome. A two-sided tested p-value ≤0.05 was considered statistically significant. The analyses were performed using SPSS Statistics software version 22.0 (IBM, Armonk, NY, USA).

### Interpretation of the results

PRO’s were interpreted by two important tools: the minimal clinically important difference (MCID) and Cohen’s standardized effect size. The MCID is defined as the smallest difference in a score which patients perceive as beneficial and which would mandate a change in patient management. For the SF-36 the MCID ranges between 3–5 points [23, 24]. Standardized effect size measures are helpful when the metrics of variables that are studied have no intrinsic meaning. The Cohen’s standardized effect size is defined as the mean difference divided by the SD. Effect sizes of 0.2–0.5 are considered as ‘small’, 0.5–0.8 as ‘moderate’, and > 0.8 ‘large’ [25].

### Ethical considerations

Oral informed consent was obtained. The medical ethical committee CMO Arnhem—Nijmegen concluded that this research is exempt from Dutch national legislation for medical scientific research involving humans in view of the minimal burden of two questionnaires.

## Results

### Participants

We identified 301 patients with proven angiodysplasias of which 69 were excluded based on: deceased at the time of survey (n = 63), severe dementia (n = 4), severe psychiatric disorder (n = 1) and physical inability due to severe Parkinson disease (n = 1). We failed to establish contact with 39 eligible and 18 patients did not want to participate. In total 175 patients gave informed consent and 155 patients returned the questionnaires (S1 Fig). Eleven SF-36 questionnaires had to be excluded due to >50% missing, and one more could not be included for MFI-20 analysis. Therefore, 144 (response rate 62%) patients are analyzed for the SF-36 and 143 (61%) for the MFI-20 (S1 Dataset).

Disease severity subgroups consisted of patients treated (n = 27), not treated (n = 58), and patients without recent hospital visits (n = 45). Fourteen patients could not be included in the subgroup analysis due to follow-up in another hospital. Additional subdividing led to 39 non-treated patients without anemia and 19 (41% of the patients with anemia) non-treated patients with anemia.

### Baseline characteristics

The majority of responders are men (65%) and the mean age was 68 years. Gender, valvular heart disease, anticoagulant use and multiple angiodysplasia lesions were not equally distributed between subgroups (Table 1). These differences reflect that these variables constitute risk factors for symptomatic angiodysplasia disease [1, 17]. There were no significant differences between the subgroups in ischemic heart disease, diabetes mellitus, chronic obstructive pulmonary disease, liver cirrhosis, congestive heart failure, or cerebral vascular accident. Angiodysplasias were most frequently located in the colon (n = 90, 69%) with a mean number of 2.4 (SD 3.2) angiodysplasias per patient.

**Table 1:** Descriptive data of all responders and comparison of asymptomatic versus symptomatic responders.

Baseline characteristics	No hospital vistis	Non-treatment	Treatment	*P*-value
Age—mean (±SD)	66yr (±10)	67yr (±11)	71yr (±9)	0.14
Gender: male—n (%)	33 (73)	38 (66)	12 (44)	0.04
Comorbidity—n (%)				
*Valvular heart disease*	5 (11)	4 (7)	7 (26)	0.04
*Chronic kidney disease*	3 (7)	7 (12)	5 (19)	0.31
Anticoagulant use—n (%)	13 (29)	18 (31)	19 (70)	<0.01
Number of angiodysplasias -mean (±SD)	1.5 (0.9)	1.9 (1.2)	5.3 (6.1)	<0.01
Angiodysplasia location				
*Colon*	36 (80)	35 (60)	19 (70)	0.10
*Small bowel*	5 (11)	12 (21)	8 (30)	0.14
*Stomach*	3 (7)	8 (14)	3 (11)	0.51
**Total**	**n = 45**	**n = 58**	**n = 27**	

### Primary outcome

**Fig 1:**
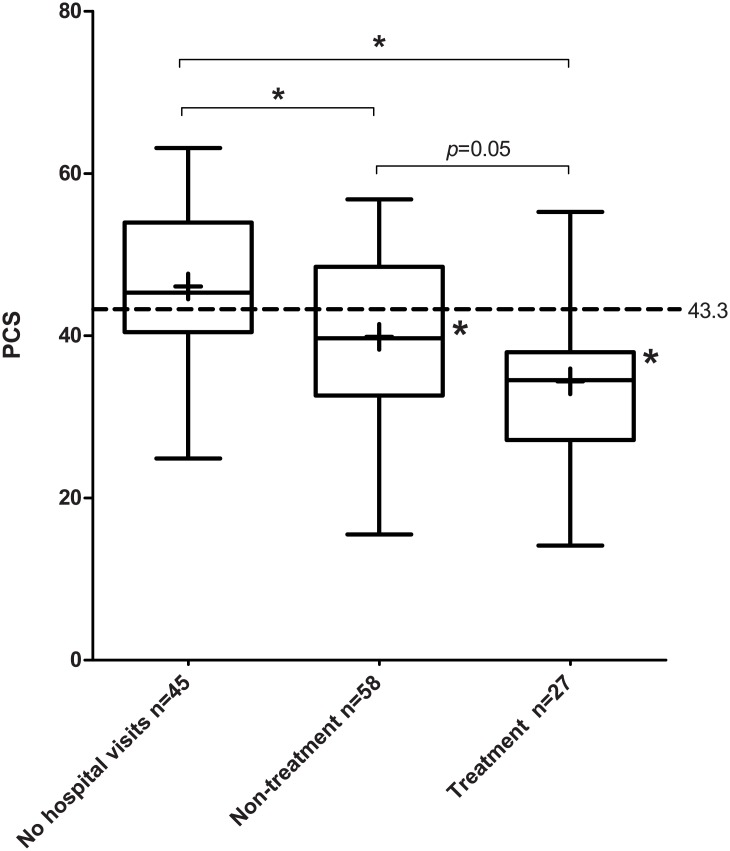
Comparison of SF-36 physical component scale (PCS) for subgroups. Boxes with whiskers display median and interquartile percentiles of subgroups, + sign indicates mean. Dotted line represents mean general population. Asterisks point out significant differences (p<0.05).

Our cohort of angiodysplasia patients (n = 144) have a significant lower PCS score relative to the general population (respectively mean 41.0 vs. 43.3, p = 0.01). However, this difference does not reach MCID and has a small effect size (effect size: 2.3/10.8 = 0.21). Focusing on the three subgroups (Fig 1), we see this is based on a significant lower PCS in the non-treatment and treatment group compared to the general population (respectively 39.9 vs. 43.3, p = 0.01 and 34.4 vs. 43.3, p<0.01) as the patients without hospital visits have a non statistical significant higher PCS score than the general population (46.1 vs. 43.3, p = 0.06). The differences between the non-treated and treated patients and the general population for PCS (resp. 3.4 and 8.9 points) exceeds the MCID threshold with a respectively small (3.4/10 = 0.34) and large effect size (8.9/10.3 = 0.86), which suggest that it bears clinical relevance.

When we adjust the primary analysis of PCS for differences in baseline characteristics, the variables age, gender, valvular heart disease, anticoagulant use and number of angiodysplasias are included in the multivariate linear regression model. The association of disease severity covered in the subgroups treated, not treated and no recent hospital visits, is despite correction of these factors independently negatively associated with PCS (ß -4.6, 95% CI -7.8–-1.3, p = 0.006). However, the negative impact of angiodysplasia disease becomes smaller than in univariate linear regression due to the influence of the comorbidities (ß -5.9, 95% CI -8.2–-3.5). The interpretation of the beta coefficient is that the treated group in general scores -4.6 points lower on PCS as the non-treated group, which scores -4.6 points worse than the no-hospital visits group.

Comparing the three subgroups with each other, the no-hospital visits patients have a significant higher PCS score compared to the non-treatment and treatment groups (respectively 46.1 vs. 39.9 and 34.4, p = 0.01 and p<0.01). However, the PCS of the non-treatment group is not statistically significant higher than the treated patients (39.9 vs. 34.4, p = 0.051).

### Secondary outcomes

#### Subdomain analysis for QoL

The study population of all angiodysplasia patients (n = 144) have compared to the general population a significant lower score on the following QoL subdomains: MCS (48.2 vs. 52.7, p<0.01), RP (40.8 vs. 44.0, p<0.01), GH (39.7 vs. 47.3, p<0.01), SF (43.7 vs. 47.6, p<0.01), RE (44.2 vs. 48.1, p<0.01) and MH (47.6 vs. 51.8, p<0.01). No significant differences were found in PF, BP and VT.

**Fig 2:**
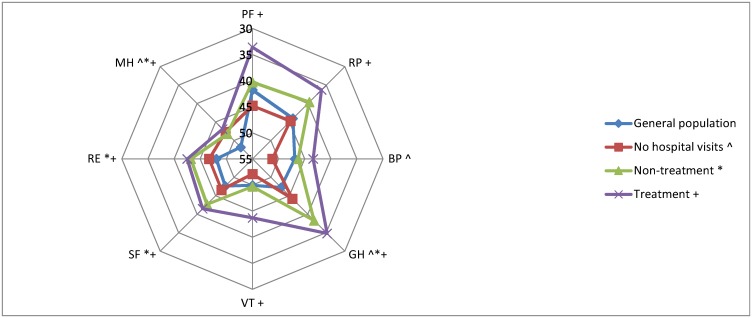
Display of all SF-36 domain scores for the different subgroups. Physical functioning (PF), Role limitations caused by Physical health problems (RP), Bodily pain (BP), General health (GH), Vitality (VT), Social functioning (SF), Role limitations caused by Emotional health problems (RE) and Mental health (MH). Statistical significant (p<0.05) differences compared to general population are shown for each subgroup with the following symbols. No hospital visits: ^, non-treatment: *, treatment: +.

Comparing the subgroups separately with the general population, we again see the differences for the overall angiodysplasia group are mainly based on the non-treatment and treatment subgroups, with the non-hospital visit group continuously scoring higher on the 8 subdomains (Fig 2). The non-treatment group have a significantly lower score compared to the general population on GH (47.2 vs. 38.3, p<0.01), SF (47.6 vs. 42.7, p<0.01), RE (48.0 vs. 43.2, p<0.01), MH (51.8 vs. 48.2, p = 0.02). As last, the treatment group have a significantly lower score compared to the general population on PF (41.7 vs. 33.6, p<0.01), RP (41.7 vs. 36.3, p = 0.02), GH (47.2 vs. 34.8, p<0.01), VT (49.9 vs. 43.7, p<0.01), SF (47.6 vs. 41.5, p = 0.01), RE (48.0 vs. 42.5, p = 0.02), MH (51.8 vs. 46.9, p<0.01).

Comparing the three subgroups there are larger differences in scores assessing physical subdomains such as PF, RP and GH (Fig 2), with the treated group scoring worse.

#### Level of fatigue

**Fig 3:**
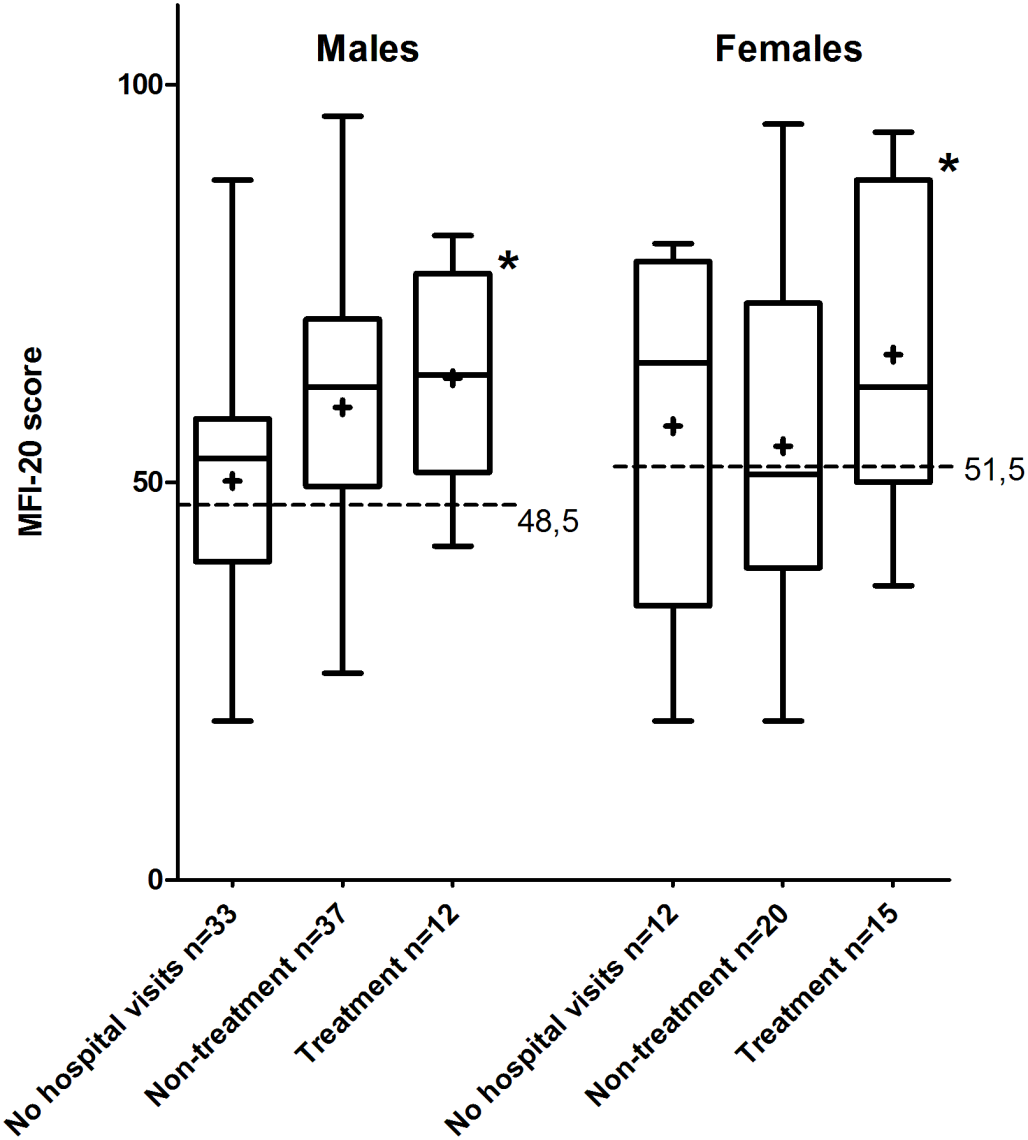
Comparison of MFI-20 scores for males (left) and females (right). Boxes with whiskers display median and interquartile percentiles of subgroups, + sign indicates mean. Dotted line represents mean general population. Asterisks point out significant differences (p<0.05).

Angiodysplasia patients have significant higher levels of fatigue compared to the general population (respectively male 56.1 vs. 48.5, p<0.01, female 59.2 vs. 51.5, p = 0.01). This difference has a small effect size (Fig 3: men = 0.43 and women = 0.39). The treatment subgroup is the only subgroup with a significant higher level of fatigue compared to the general population for males (resp. 63.1 vs. 48.5, p<0.01) and females (resp. 66.1 vs. 51.5, p = 0.01). This difference has a large effect size (men = 1.16 and women = 0.76).

#### Additional non-treatment subgroup analysis QoL

**Fig 4:**
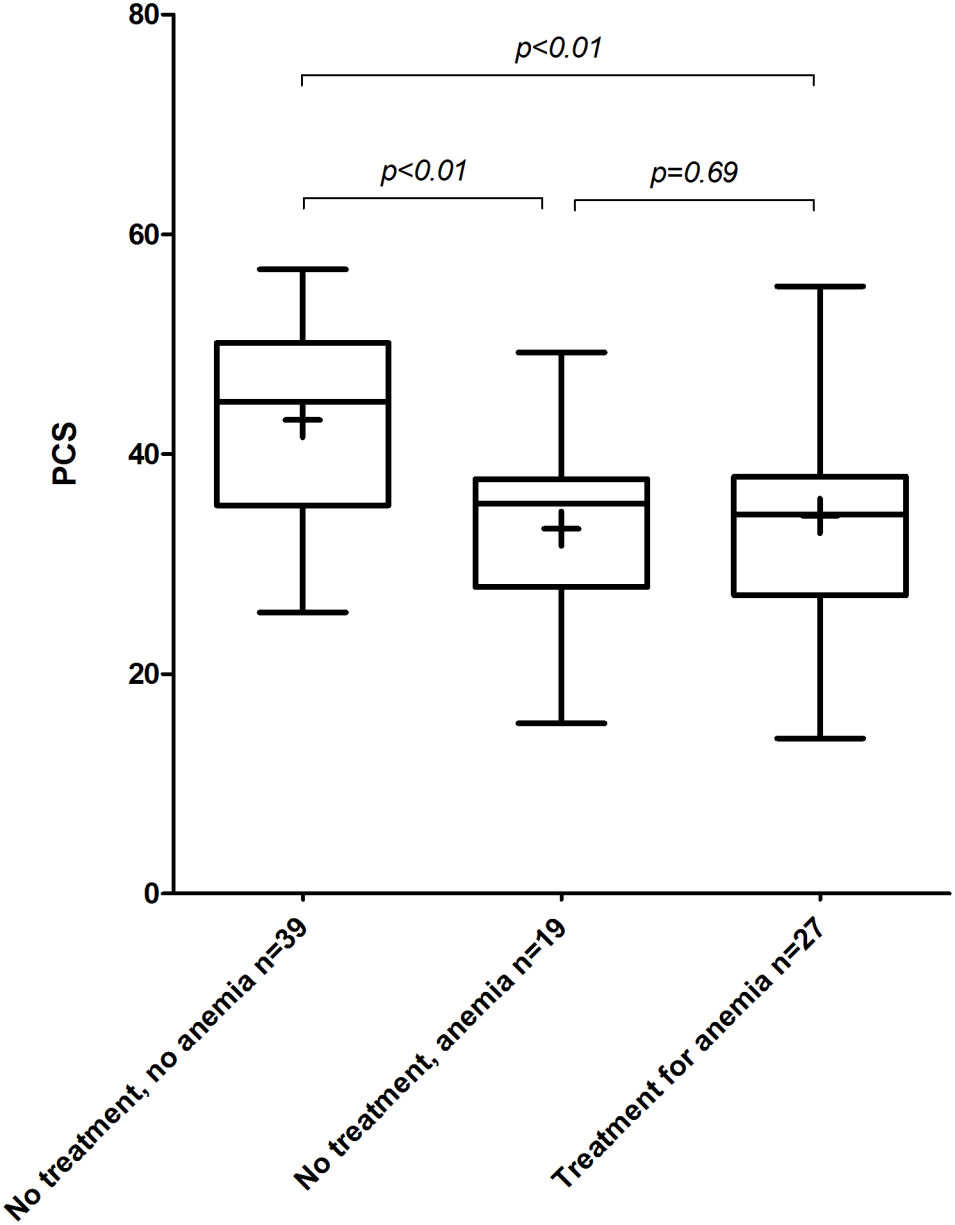
Comparison of SF-36 Physical component scale (PCS) for disease severity subgroups. Boxes with whiskers display median and interquartile percentiles of subgroups, + sign indicates mean.

To investigate the effect of anemia in the non-treated group on the QoL, the non-treated group was dichotomized according to presence of anemia (Fig 4). The patients without anemia have a significantly higher PCS score compared to the non-treated patients with anemia and the treated patients (resp. 43.1 vs. 33.2 and 34.4, p<0.01 and p<0.01).

## Discussion

The results of this cross-sectional study show that the presence of GI angiodysplasias is associated with a decreased QoL compared to the general population. Angiodysplasia patients have a lower self-rated QoL and higher fatigue levels, and particularly the physical component of QoL is significantly reduced. Moreover, we found a progressive decrease in QoL that corresponds with disease severity. Finally, the QoL between treated patients and those not treated with anemia is similar. This supports the relevance of a structured approach and suggest that iron supplementation in patients with angiodysplasias and mild anemia is warranted. Treatment of mild anemia in angiodysplasia disease is often withheld (41%).

To investigate whether a decreased QoL in our study population depends on co-existing co-morbidity, we performed a multivariate regression analysis that corrected for differences in co-morbidities at baseline. It was clear that angiodysplasia disease severity is an independent variable that impacts health-related QoL.

The decrease in PCS associated with angiodysplasias is comparable to that seen in other populations such as the elderly with anemia, [8] congestive heart failure or patients with anemia due to chronic kidney disease [26]. This indicates that anemia in symptomatic angiodysplasias impacts QoL to a similar level as other chronic diseases. The MCID data in relation to the effect sizes suggests that our findings bear clinical relevance.

The present study has several limitations. First, this is a single center study with retrospective data collection of the clinical data, e.g. medical history and treatment. This could introduce reporting bias for the variables that have been retrospectively assessed (i.e. not our primary endpoint). Second, SF-36 and MFI-20 are tools to measure health-related QoL and level of fatigue respectively. Addition of the Functional Assessment of Cancer Therapy-Anemia (FACT-An) questionnaire, specific for anemia would have been desirable. However, this tool is not available in a validated Dutch version. Third, the definitions used to classify the disease severity subgroups (i.e. treatment in the last year) are based on consensus meeting in view of the absence of published criteria. Fourth, categorizing of our cohort in subgroups resulted in a small number of patients compromising the study power. Last, similar to all questionnaire studies response bias could be present. We have a response rate of 62% which is relatively high [27–29]. Age, gender and past medical history were compared between responders and non-responders to investigate differences that could have affected the results. Responders were more often men than non-responders (respectively 64% vs. 49%, p = 0.02). Men tend to score better on QoL questionnaires and therefore we assume this could have led to a small overestimation of the QoL.

The strength of this study is that it measured QoL in angiodysplasia patients in a relatively large cohort with the possibility to compare different disease severities. Moreover, our study population was a heterogeneous angiodysplasia population. The risk of selection bias was low due to a relatively high response rate and absence of clinical relevant differences between responders and non-responders. Therefore, we think this study has a high external validity. With a multivariate model we were able to correct for baseline differences in patient characteristics between the disease severity subgroups, which increases the internal validity of this study.

The results of this study highlight the need for additional clinical trials. For instance, a study which evaluates different step-up treatment strategies in patients with angiodysplasias, anemia and a reduced QoL. For such studies to have a true impact on practicing physicians, they should reflect patient management in clinical practice. This would require a comparative trial with a sufficient number of patients using practical inclusion criteria, clear crossing-over criteria, a treatment failure scenario, and end-points that are clinically relevant in terms of PRO’s. QoL measurements should be an integral part of the study.

In conclusion, this study shows GI angiodysplasias are independently associated with clinically significant impairments in multiple domains of health-related QoL, especially in measures of functional limitation. Moreover, angiodysplasia patients experience a higher level of fatigue compared to the general population. We encourage future research investigating treatment strategies in angiodysplasias to take the patient’s perspective into account with the inclusion of PRO’s such as the SF-36.

## Supporting information

S1 Table STROBE statement. Checklist of items that should be included in reports of cross-sectional studies.
(DOCX)

S1 Fig Flowchart patient inclusion. Small-dot lined squares represent ‘non-responders’. The ‘complete returned questionnaires’ are the ‘responders’.
(TIFF)

S1 Dataset The raw dataset on which the analysis are performed. (SAV)

## Abbreviations

BP: Bodily pain
GH: General health
GI: Gastrointestinal
MCID: Minimal clinically important difference
MCS: Mental summary score
MFI-20: Multidimensional fatigue inventory
MH: Mental health
n: Number
PCS: Physical summary score
PF: Physical functioning
QoL: Quality of Life
RE: Role limitations due to emotional problems
RP: Role limitations due to physical health problems
SD: Standard deviation
SF: Social functioning
SF-36: Short Form Health Survey
VT: Vitality
